# Individuals Diagnosed with Schizophrenia Assign Emotional Importance to Neutral Stimuli: An fMRI Study

**DOI:** 10.1155/2013/965428

**Published:** 2013-12-05

**Authors:** Nadia Lakis, Adrianna Mendrek

**Affiliations:** ^1^Centre de Recherche de l'Institut Universitaire en Santé Mentale de Montréal, 7331, rue Hochelaga, Montreal, QC, Canada H1N 3V2; ^2^Department of Psychiatry, Faculty of Medicine, University of Montreal, C.P. 6128 succursale Centre-ville, Montréal, QC, Canada H3C 3J7; ^3^Department of Psychology, Bishop's University, 2600, rue College, Sherbrooke, QC, Canada J1M 1Z7

## Abstract

The majority of functional neuroimaging studies investigating neural correlates of emotion processing in schizophrenia report a significant deficit in limbic structures activation in patients relative to control participants. Recently it has been suggested that this apparent “deficit” could be due to an enhanced sensitivity of the neutral material in individuals diagnosed with schizophrenia, rather than due to their inefficiency in emotion processing. The purpose of the present study was to test this supposition and verify if the potential effect is present in both men and women diagnosed with schizophrenia. In order to do that we examined the pattern of cerebral activation associated with processing of neutral stimuli in schizophrenia. Thirty-seven schizophrenia patients and 37 healthy controls viewed neutral and emotional images while in a functional magnetic resonance imaging scanner. Schizophrenia patients rated the neutral images as more emotionally salient than controls. Additionally, patients showed significant activation during processing of neutral images in limbic and prefrontal regions; similar areas were underactivated in patients relative to controls during processing of emotional information. Investigation of sex differences revealed that the enhanced responsiveness to the emotionally neutral material was attributed primarily to men with schizophrenia.

## 1. Introduction

Functional neuroimaging studies, which explored processing of emotional material in schizophrenia, have often reported “deficits” in cerebral activation in patients compared to control participants in various limbic, paralimbic, and prefrontal regions (e.g., [1–4]). A few other studies documented “abnormal overactivation” in patients relative to controls or no difference between the groups [5–10]. These divergent findings have been attributed to the type of emotional task (passive viewing, emotion identification, emotional memory, etc.) and to the characteristics of the recruited patients (first-episode versus chronic, medicated versus unmediated, presence of prominent negative versus prominent positive symptoms, etc.). However, what could have played an equally or even more important role in the obtained results is the kind of functional neuroimaging contrast used in the statistical analysis.

It should be pointed out to readers less familiar with the functional neuroimaging literature that the functional magnetic resonance imaging (fMRI) studies in various clinical populations (including schizophrenia and related psychoses) have relied primarily on comparisons between two different states under investigation (e.g., active experimental task versus passive relaxed state) and as such fMRI is principally a relative technique with no absolute baseline. The most commonly used contrast in functional neuroimaging studies of emotions consists of subtracting brain activation associated with processing of neutral stimuli from the cerebral activity associated with processing of emotional stimuli. Another contrast routinely used is to subtract brain function linked with a resting baseline from the brain activation associated with emotional stimuli. Interestingly, in a meta-analysis examining amygdala recruitment in response to aversive emotional material in schizophrenia, Anticevic and colleagues [11] have found that underactivation of the amygdala in patients compared with controls was only present in studies that used an emotional minus neutral contrast and not present in those that employed an emotional minus rest condition. Hence, the “deficient” activation of the amygdala in patients compared to controls could have been explained by an increase of activity in this region in response to neutral stimuli. In other words, it is not a deficit in the amygdala activation, but rather its oversensitivity to the neutral material in individuals diagnosed with schizophrenia that may have produced the impression that patients do not recruit this region to the same extent as controls.

To date, only a few studies have overtly reported or explored the brain activation associated with the processing of neutral stimuli versus baseline in schizophrenia patients, including previous work by our group [12–15]. We found that while patients with schizophrenia had relative deactivation compared with controls in the middle frontal gyrus, orbitofrontal cortex, cingulate gyrus and precuneus during recognition memory of emotional relative to neutral images, they activated some of these same regions to a significantly greater degree than did the healthy controls during recognition memory of neutral pictures relative to a resting baseline [14].

Considering the methodological heterogeneity that exists in the schizophrenia literature with regard to the neural correlates of emotion processing, explicitly examining the differences in the pattern of brain activation between patients and healthy subjects in response to neutral stimuli may broaden our understanding of emotional “deficits” in this complex psychiatric disorder. Thus, the aim of the present study was two-fold: (1) to extend our previous findings and explore whether patients with schizophrenia show an atypical pattern of brain activity in response to neutral stimuli during the incidental encoding of emotional stimuli that preceded our emotional recognition memory paradigm and (2) to explore potential sex differences, as previous studies investigating processing of emotionally neutral stimuli examined exclusively or primarily men.

## 2. Methods

### 2.1. Participants

Thirty-seven individuals diagnosed with schizophrenia (19 men) according to DSM-IV diagnostic criteria [16] and 37 healthy controls (19 men) participated in the study. All patients were in a stable phase of their illness (defined as no relapse within the last two months and no change in their antipsychotic treatment within the last month). The groups were matched for age, sex, handedness (Edinburgh Inventory) [17], and parental socioeconomic status (National Occupational Classification (NOC)) [18].

All patients were reevaluated by experienced psychiatrists before being assigned to the research group (DSM-IV, criteria A-E); affective, schizoaffective, and schizophreniform psychoses were excluded. Control participants were screened with the nonpatients edition of the Clinical Interview for DSM-IV (SCID) [19]. Symptom severity was rated according to the positive and negative syndrome scale (PANSS) [20]. Illness onset was defined as the date of the first psychiatric consultation. All the patients received at least one atypical antipsychotic (chlorpromazine equivalence was calculated) [21] (27 patients received one, 9 received two, and 1 received three. Clozapine: *n* = 19, mean dosage = 452.63 (77.23) mgs; olanzapine: *n* = 12, mean dosage = 14.58 (5.4) mgs; risperidone: *n* = 11, mean dosage = 3.73 (1.67) mgs; quetiapine: *n* = 7, mean dosage = 585.71 (238.85) mgs).

General exclusion criteria included age below 18 or above 45 years, past or present neurological or Axis-I psychiatric disorder, alcoholism or drug abuse, noncompliance with testing procedures, abnormal uncorrected vision, or any contra-indication for MRI such as a cardiac pacemaker, an aneurysm clip, a metal prostheses or cardiac valve replacement, the presence of metal in an eye or any part of the body, certain dental work, or claustrophobia.

In agreement with the Declaration of Helsinki, written informed consent was obtained prior to participation in the experiment. The ability of schizophrenia patients to give informed consent was established using the guidelines of the Canadian Psychiatric Association. The study was approved by the ethics committees of the Fernand-Seguin Research Center of the Louis-H Lafontaine Hospital and the Regroupement Neuroimagerie Québec. Full details of subject characteristics are given in Table 1.

### 2.2. Experimental Procedure and Design

Participants passively viewed blocks of positive, negative, and neutral pictures while in the fMRI scanner. Each block was 48.5 seconds in length and there were 16-second periods of rest separating the blocks from one another. Each block contained 10 images and there were 4 blocks in each experimental condition. Each picture appeared for 3000 ms followed by a blank screen with a fixation point. To assess the participants subjective emotional responses to the presented images, participants were represented with the images of each block at the end of the fMRI session and were asked to rate the block of images as whole on a scale ranging from 0 (absence of any emotional reaction) to 8 (strongest emotion ever felt in one's lifetime) the intensity of experienced emotion.

### 2.3. fMRI Data Acquisition, Processing, and Analysis

Blood oxygenation level dependent (BOLD) signals were recorded using a single-shot, gradient-recalled echoplanar imaging sequence (repetition time (TR) = 3000 ms, echo time (TE) = 30 ms, flip angle = 90 degrees, and matrix 64 × 64 voxels) on a MRI Siemens TRIO system at 3.0 Tesla, which is operational at the Functional Neuroimaging Unit at the University of Montreal Geriatric Institute. The functional volumes were then registered to individual high-resolution coplanar anatomical images taken during the same scanning session (three-dimensional, spoiled gradient echosequence; 28 slices, slice thickness = 5 mm, TR = 22 ms, and TE = 4 ms, and flip angle = 30′′; matrix 256 × 256 voxels) to better identify activated structures.

The fMRI data was analyzed using statistical parametric mapping software (SPM5; Wellcome Department of Cognitive Neurology, London, UK) according to the methods outlined by Friston [22]. Functional images were realigned to the mean volume of each session to correct for artifacts due to subject motion, were spatially normalized into the standardized brain template (voxel size: 3.5 mm × 3.5 mm × 3.5 mm), and were spatially smoothed with a three-dimensional isotropic Gaussian kernel (12 mm FWHM) to improve the signal-to-noise ratio.

Statistical analyses were carried out using a standard peak-detection approach and the general linear model implemented in SPM5 to identify the dynamic cerebral changes associated with the processing of emotional material. First, fMRI data of each participant was analyzed using a fixed-effects model to investigate individual brain activation maps and to contrast the brain activity associated with different conditions. The fixed-effects analysis produced individual contrast images that were then used as raw data for the implementation of a random-effects model to investigate the pattern of activation during the different emotional contrasts (i.e., NEG versus NTR, POS versus NTR, and NTR versus REST) in each group (i.e. healthy controls and schizophrenia patients). Any potential differences between groups were examined using a two-sample *t*-test. Due to the strict character of the second-level analysis based on a random-effects model, the statistical maps were thresholded at a level of *P* = 0.005 uncorrected for multiple comparisons. The centers for each of our a priori ROIs were produced using the Mask for ROI Analyses software (MARINA) [23]. This software provides 3D masks based on the Automated Anatomical Labeling (AAL) [24]. AAL uses the anatomical boundaries of each region using the MNI template as a reference. A search sphere with a radius of 12 mm was applied to the center of the hippocampus using the small volume correction function in SPM5. For the amygdala, a sphere of 8 mm was implemented. The AAL tool in SPM provided the anatomic labeling of each activation peak within the ROI. For the a priori search, a probability threshold for multiple comparison of a corrected *P* < 0.05 and a *z*-score 1.67 was used [25]. Effects at each voxel of the brain were estimated using the general linear model and voxel values for the contrasts of interest generated statistical parametric maps of the *t* statistic (SPM *t*) that were subsequently transformed to the unit normal distribution (SPM *Z*).

The demographic, clinical, and behavioral data were analyzed with the Statistical Package for the Social Sciences (SPSS), version 15.0.

## 3. Results

### 3.1. Behavioral-Subjective Ratings

The ANOVA revealed a significant effect of group (*F*(3) = 3.75, *P* = 0.015) but no significant effect of sex (*F*(3) = 0.94, *P* = 0.43) or sex by group interaction (*F*(3) = 1.39, *P* = 0.25) (Table 1). Posthoc *t*-tests revealed no significant difference in the way patients and controls rated the emotional images (*P* > 0.05), while schizophrenia patients rated the neutral images with a greater emotional intensity than did the healthy subjects (healthy: mean = 1.08  (0.97), schizophrenia: mean = 1.74  (1.38), *P* = 0.027).

### 3.2. fMRI-Emotion Processing, Neutral Processing, and Sex Differences in Neutral Processing

**Figure 1:**
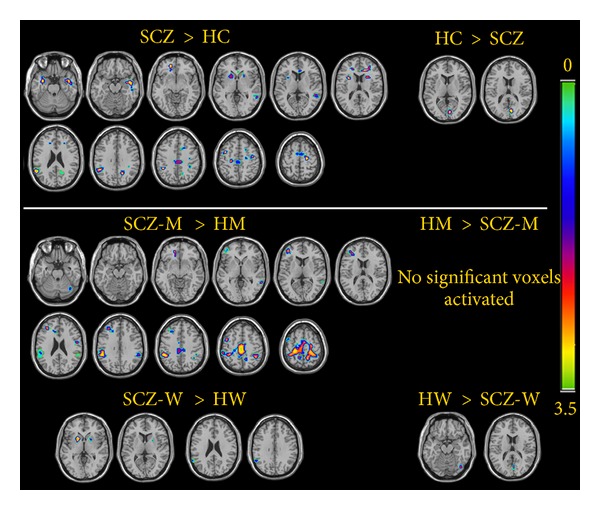
Group (top) and sex (bottom) differences in cerebral activation associated with processing of neutral material in healthy controls and schizophrenia patients. HC: healthy controls; SCZ: schizophrenia patients; HM: healthy men; SCZ-M: schizophrenia men; HW: healthy women; and SCZ-W: schizophrenia women. The colored bar represents the range of *z*-scores of the cerebral activation with significance increasing from 0 (green-blue color) to 3.5 (yellow-green color).

Detailed information regarding functional neuroimaging data (brain regions with Brodmann areas, MNI coordinates, number of activated voxels in a given cluster, *z*-scores, *P* values, etc.) is provided in the tables and figures; for brain regions activated during emotion processing refer to Table 2; for brain regions activated during processing of neutral stimuli refer to Table 2 and Figure 1; for sex differences in cerebral activation during processing of neutral stimuli refer to Table 3 and Figure 1.

## 4. Discussion

Consistently with numerous previous studies investigating neural correlates of emotion processing in schizophrenia, which involved diverse paradigms ranging from discrimination of emotional stimuli [1, 26], happy, and sad mood induction [3, 27] and experience of various emotional states [2, 4, 28], to passive viewing of emotional stimuli [29], in the present study, we observed relatively less activation in patients versus controls in the brain regions typically associated with emotion processing in the general population. This “deficit” was apparent when making a classic comparison between processing of emotional versus neutral stimuli and should lead us to conclude that individuals diagnosed with schizophrenia cannot properly activate their limbic system structures. Importantly however, we have also made a comparison between the neutral stimuli and simple “rest” and found that under these circumstances it was the patients who activated several brain regions to a greater extent than did comparison participants. This pattern of functional neuroimaging results was complemented by the subjective rating data, which showed an attribution of greater emotional salience to neutral stimuli in patients relative to controls.

A few investigations of processing of emotionally neutral stimuli in schizophrenia patients have documented increases in the amygdala, hippocampus, parahippocampal, posterior cingulate, and fusiform gyrus [12, 13, 15] in response to faces with neutral expressions. Our current findings support and extend these previous results in showing increased activity in the amygdala and fusiform gyrus in addition to other brain regions implicated in affect, including the middle and anterior cingulate gyrus, middle temporal pole, middle temporal gyrus, and orbitofrontal cortex [30]. The middle frontal and parietal cortex, also activated to a greater extent in patients relative to controls during neutral versus rest condition, has been found to play a more general role in nonemotional episodic memory [31, 32]. Although the participants were not told to try and remember the images, they knew that a memory task would follow the initial encoding phase of the study and so it is feasible that, relative to controls, schizophrenia patients made a greater implicit (or explicit) effort to remember the stimuli.

Previous studies investigating responses to neutral stimuli in schizophrenia used samples consisting exclusively or primarily of male patients (and controls), rendering it impossible to verify whether the same pattern of subjective experience and cerebral activation would be also present in women with schizophrenia. Because in the present study we included a sufficiently large number of male and female participants, we could make sex-specific comparisons. Thus, we observed that relative to the same-sex controls, men with schizophrenia activated a significantly larger neural network that included the cingulate gyrus, as well as prefrontal and parietal cortices, while only a slight difference in cerebral activation was found between the two groups of women. Thus, the difference in the processing of emotionally neutral material found between all the participating patients and the comparison group appeared to be driven mainly by men. This finding emphasizes the need to analyze data of the two sexes separately in the studies of emotional processing and cognitive function in schizophrenia and related psychosis, as noted elsewhere [33, 34].

An interesting model of the origin of psychosis is that it is characterized by attribution of emotion to external and internal events, which are typically considered neutral [35, 36]. Thus, the increased neural response to neutral stimuli in our schizophrenia sample may reflect an atypical assignment of motivational salience to the neutral pictures—a premise reinforced by the fact that the schizophrenia patients attributed greater emotional importance to the neutral images than did the healthy subjects. An interesting topic of debate is whether this abnormal pattern of activity reflects a trait or state marker of the disorder. Seiferth and colleagues [37] reported increased neural responses to neutral faces in persons at risk for psychosis. Nonetheless, evidence has also been put forth emphasizing the potential role of clinical symptoms on this interesting pattern of activity. For instance, some have reported a significant positive relationship between positive symptoms and brain activation associated with nonemotional stimuli in patients with schizophrenia [15, 38], as well as increased visual attention to neutral but not threatening components of social scenes in patients with persecutory delusions [39]. More research in this area, particularly in populations at high risk for developing schizophrenia, is needed to fully understand the neural response to neutral material.

## 5. Conclusions

The present study supports and extends a few previous reports of enhanced neural and subjective sensitivity to the emotionally neutral material in individuals diagnosed with schizophrenia. Importantly, we have shown for the first time that this effect is present primarily in male patients, while women with schizophrenia appear more similar to the same-sex control participants in this particular context. More studies are warranted to explore this effect further.

## Figures and Tables

**Table 1:** Demographic and clinical data of participants.

	Schizophrenia patients		Healthy controls		*P* (two-tailed)
	Mean	SD	Mean	SD	
Age	32.46	7.66	31.81	6.91	0.70
Parental SES	2.36	1.09	2.68	0.88	0.18
Sex (% male)	51	—	51	—	—
Handedness (% right)	89	—	91	—	—
Medication (chlorpromazine equivalence)	613.92	361.02	—	—	—
PANSS positive	18.84	6.93	—	—	—
PANSS negative	12.59	10.95	—	—	—
PANSS general	24.05	22.59	—	—	—
Subjective ratings—positive	5.77	1.20	5.36	1.21	0.16
Subjective ratings—negative	6.67	1.28	6.94	0.95	0.32
Subjective ratings—neutral	1.74	1.38	1.08	0.97	0.027*

SES: socioeconomic status; SD: standard deviation.

*The value is statistically significant.

**Table 2:** Brain regions activated in healthy controls and schizophrenia patients.

Group	L/R	Brain region	BA	MNI coordinates			*Z*-score	Voxels	*P* value
				*X*	*Y*	*Z*			
Negative minus neutral									
HC minus SCZ	L	Middle temporal gyrus	20	−52	−35	−14	2.74	237	0.003
	R	Anterior temporal pole	38	56	10	−18	2.56	164	0.005
	L	Cerebellum	—	−7	−46	−49	2.56	185	0.005

Positive minus neutral									
HC minus SCZ	R	Posterior cingulate gyrus	29	4	−42	14	3.06	190	0.001
	L	Middle cingulate gyrus	23	−10	−35	32	2.76	*	0.003
	L	Hippocampus	—	−35	−35	−4	2.68	30	0.04
	R	Amygdala	—	32	0	−24	2.79	9	0.017
	L	Middle temporal gyrus	20	−42	0	−28	2.74	19	0.003
	L	Precuneus	7	−13	−63	38	2.63	25	0.004

Neutral minus rest									
HC minus SCZ	L	Calcarine gyrus	17	−7	−84	7	3.07	32	0.001
SZ minus HC	R	Angular gyrus	40	56	−52	24	3.19	65	0.001
	L	Middle temporal gyrus	20	−49	−10	−24	3.13	79	0.001
	L	Fusiform gyrus	20	−38	−10	−21	2.96	*	0.002
	R	Superior OFC	10	14	46	−14	3.06	22	0.001
	L	Hippocampus	—	−32	−7	−21	2.74	8	0.045
	R	Amygdala	—	32	0	−28	3.01	11	0.006
	R	Middle temporal pole	38	38	10	−32	2.76	*	0.003
	L	Middle frontal gyrus	46	−32	32	18	2.92	43	0.002
	L	Anterior cingulate gyrus	24	−7	33	10	2.78	*	0.003
	R	Middle cingulate gyrus	31	4	−28	42	2.92	53	0.002
	L	Middle cingulate gyrus	32	−10	10	35	2.69	13	0.004
	L	Superior frontal gyrus	8	−18	18	42	2.68	*	0.004
	R	Anterior cingulate gyrus	32	14	36	14	2.74	11	0.003
	L	Supplementary motor area	6	−4	−4	63	2.83	18	0.002
	L	Inferior parietal gyrus	40	−35	−42	35	2.67	6	0.004
	L	Cuneus	7	−18	−60	28	2.96	20	0.002
	R	Precentral gyrus	6	42	0	46	2.94	15	0.002
	R	Putamen	—	32	7	10	3.05	17	0.001
	L	Putamen	—	−21	10	−4	2.65	5	0.004

BA: Brodmann area, HC: healthy controls, SCZ: schizophrenia patients, OFC: orbitofrontal cortex; *brain region part of the same cluster.

**Table 3:** Brain regions activated in healthy and schizophrenia men and women.

Group	L/R	Brain region	BA	MNI coordinates			*Z*-score	Voxels	*P* value
				*X*	*Y*	*Z*			
Neutral minus rest									
HM minus SCZ-M	No significant voxels activated								
SCZ-M minus HM	R	Middle cingulate gyrus	31	4	−24	46	3.52	1014	0.001
	L	Superior frontal gyrus	6	−18	−10	74	2.92	*	0.002
	L	Inferior parietal gyrus	40	−35	−46	52	3.00	*	0.001
	R	Inferior parietal gyrus	40	35	−42	52	3.05	*	0.001
	L	Supplementary motor area	6	0	−4	63	3.15	*	0.001
	L	Precuneus	7	−10	−56	52	3.15	*	0.001
	R	Anterior cingulate gyrus	32	10	24	21	2.72	6	0.003
	L	Cerebellum	—	−14	−84	−35	3.26	179	0.001
	R	Cerebellum	—	10	−80	−42	2.75	8	0.003
	R	Precentral gyrus	6	49	0	49	3.00	22	0.001
	R	Middle frontal gyrus	10	42	46	4	2.98	94	0.001
	R	Superior OFC	11	18	42	−14	2.92	15	0.002
	L	Middle temporal gyrus	21	−60	−49	0	2.89	9	0.002
	L	Supramarginal gyrus	40	−56	−46	28	2.81	13	0.002
HW minus SZC-W	L	Inferior occipital gyrus	19	−42	−74	−14	2.77	9	0.003
	L	Calcarine gyrus	17	−4	−80	10	2.66	5	0.004
SCZ-W minus HW	R	Putamen	—	21	7	−4	3.00	25	0.001
	L	Putamen	—	−18	7	0	2.76	6	0.003
	R	Angular gyrus	40	56	−56	28	2.79	7	0.003

BA: Brodmann area, HM: healthy men, HW: healthy women, SCZ-M: schizophrenia men, SCZ-W: schizophrenia women, OFC: orbitofrontal cortex; *brain region part of the same cluster as above.
